# Defective Pulmonary Innate Immune Responses Post-Stem Cell Transplantation; Review and Results from One Model System

**DOI:** 10.3389/fimmu.2013.00126

**Published:** 2013-05-24

**Authors:** Racquel Domingo-Gonzalez, Bethany B. Moore

**Affiliations:** ^1^Graduate Program in Immunology, University of Michigan, Ann Arbor, MI, USA; ^2^Division of Pulmonary and Critical Care Medicine, Department of Internal Medicine, University of Michigan, Ann Arbor, MI, USA; ^3^Department of Microbiology and Immunology, University of Michigan, Ann Arbor, MI, USA

**Keywords:** pulmonary complications, hematopoietic stem cell transplantation, eicosanoids, alveolar macrophage, polymorphonuclear leukocytes, scavenger receptors, microRNA, prostaglandins E

## Abstract

Infectious pulmonary complications limit the success of hematopoietic stem cell transplantation (HSCT) as a therapy for malignant and non-malignant disorders. Susceptibility to pathogens in both autologous and allogeneic HSCT recipients persists despite successful immune reconstitution. As studying the causal effects of these immune defects in the human population can be limiting, a bone marrow transplant (BMT) mouse model can be used to understand the defect in mounting a productive innate immune response post-transplantation. When syngeneic BMT is performed, this system allows the study of BMT-induced alterations in innate immune cell function that are independent of the confounding effects of immunosuppressive therapy and graft-versus-host disease. Studies from several laboratories, including our own show that pulmonary susceptibility to bacterial infections post-BMT are largely due to alterations in the lung alveolar macrophages. Changes in these cells post-BMT include cytokine and eicosanoid dysregulations, scavenger receptor alterations, changes in micro RNA profiles, and alterations in intracellular signaling molecules that limit bacterial phagocytosis and killing. The changes that occur highlight mechanisms that promote susceptibility to infections commonly afflicting HSCT recipients and provide insight into therapeutic targets that may improve patient outcomes post-HSCT.

## Hematopoietic Stem Cell Transplantation Overview

Early observations made in the 1950s paved the way for the development of hematopoietic stem cell transplantation (HSCT). Lorenz (1951) showed that animals exposed to radiation could be protected by infusion of bone marrow cells. These studies, together with the previous observation that the deleterious effects of ionizing radiation could be prevented in mice by shielding organs with lead, pushed transplantation forward as a possible therapy for patients suffering from hematologic malignancies (Little and Storb, 2002). HSCT is now used to treat a variety of disorders, including malignant, congenital, and autoimmune disorders (Cutler, 2005; Copelan, 2006).

Prior to receiving a graft, transplant recipients undergo conditioning regimens that include total body irradiation (TBI), chemotherapy, or a combination of TBI and chemotherapy (Aschan, 2007; Passweg, 2012). These regimens subject the recipients’ organs, including the lungs, to their cytotoxic effects. The hope is to eradicate the malignant or autoimmune cells and replenish the patient with healthy hematopoietic stem cells (HSCs). If the intensity of the conditioning regimen is myeloablative, repopulation of hematopoietic cells will predominantly come from the donor HSCs (Tomita, 1994; Aschan, 2007). Non-myeloablative conditioning, also known as reduced intensity conditioning (RIC) will allow repopulation from both donor and host HSCs. Depending on the underlying disease, HSCT recipients receive self HSCs (autologous) or HSCs from a human leukocyte antigen (HLA)-matched, related, or unrelated donor (allogeneic). A recent study analyzing the global use of transplantation reported that autologous HSCT continues to be more highly implemented than allogeneic transplant (Gratwohl et al., 2010). Although autologous transplant recipients are unlikely to suffer from graft-versus-host disease (GvHD) which arises when the donor leukocytes mount an immune response against different HLA antigens expressed by the host, autologous transplants performed for hematologic malignancies lack the beneficial effects of graft-versus-leukemia (GvL). In the GvL reaction the allogeneic donor HSCs recognize and remove leukemic cells within the host by virtue of the HLA mismatches (Kolb, 2008).

Traditionally, HSCs were aspirated from the bone marrow of donor patients and later infused into recipients. This procedure was often painful for the donor. However, technical advances now allow for mobilization of HSCs into the peripheral blood with the use of the growth factor, granulocyte-colony stimulating factor (G-CSF) (Cutler, 2005; Copelan, 2006). Treatment of donors with G-CSF allows HSCs to be collected with a simple blood draw. Additionally, HSCs can also be harvested from umbilical cord blood (Tse, 2008; Peffault de Latour et al., 2013). Each of these techniques has inherent advantages and disadvantages and have been discussed elsewhere (Körbling and Anderlini, 2001; Copelan, 2006; Tse, 2008). RIC has evolved as an alternative to the more traditional myeloablative conditioning regimens for individuals that would have otherwise been unable to undergo transplantation as a therapy due to age, comorbidities, or disorders associated with high-risk non-relapse mortality (Giralt, 2005). Interestingly, despite a reduction in intensity regimens, recipients of RIC HSCT display similar incidence of infectious complications and infection-related mortality (Kim et al., 2013).

## Pulmonary Complications

Although HSCT has become standard care for malignant and non-malignant disorders, its success is significantly limited by the different pulmonary complications that arise following transplantation (Cordonnier, 1986; Griese, 2000; Roychowdhury, 2005; Afessa and Peters, 2006; Copelan, 2006). The engraftment process can be characterized by three distinct phases: the pre-engraftment (0–30 days), early post-engraftment (30–100 days), and late post-engraftment phases (beyond 100 days) (Afessa and Peters, 2006; Wingard et al., 2010; Coomes et al., 2011). HSCT recipients are more susceptible to infections prior to engraftment and early after engraftment. During this early time period, the increased susceptibility is mostly due to the incomplete recovery of the immune cell compartment, and is often associated with decreased neutrophil numbers or function (Zimmerli et al., 1991). Although engraftment indicates the transplant was successful in reconstituting hematopoietic cell populations, it is evident that this does not correlate with recovery of an effective immune system, as patients remain susceptible to diverse complications (including infections) long after engraftment occurs (Soubani, 1996; Afessa and Peters, 2006; Coomes et al., 2011). Pulmonary complications, both infectious and non-infectious, have been reported to affect up to 60% of transplant recipients (Soubani, 1996; Copelan, 2006). Similarly, autopsy studies performed on both allogeneic and autologous transplant recipients revealed pulmonary complication rates of 80–89% (Roychowdhury, 2005). Although allogeneic transplants have increased incidence of infectious pulmonary complications, this may be attributed, in part, to the use of immunosuppressive therapy required to prevent GvHD. Additionally, GvHD can cause mucosal barrier disruptions which also increase susceptibility to pathogens. Interestingly, however, autologous HSCT recipients also continue to suffer from increased susceptibility to pulmonary infections even though this form of transplant does not require immunosuppressive therapies (Afessa et al., 2012). These observations highlight the inherent dysfunction of the immune response following transplant and suggest that some degree of impaired function is independent of the immunosuppressive therapies or GvHD. The idea that alveolar macrophages (AMs) are inherently less functional post-HSCT is supported by previous observations that AMs obtained from pulmonary lavage of allogeneic HSCT patients late in the post-engraftment phase were functionally defective even in the absence of overt pulmonary infection (Winston et al., 1982). Defects noted in the AMs from these patients included defects in chemotaxis, phagocytosis, and killing of *Candida pseudotropicalis* as well as phagocytosis and killing of bacteria including *Staphylococcus aureus* and *Listeria monocytogenes*. This same study suggested that the defects may be primarily confined to the lung as the function of peripheral blood monocytes in these same assays was similar to control patients (Winston et al., 1982).

Non-infectious complications that arise following transplant include peri-engraftment respiratory distress syndrome (PERDS), diffuse alveolar hemorrhage (DAH), idiopathic pneumonia syndrome (IPS), and bronchiolitis obliterans syndrome (BOS) (Soubani, 1996; Afessa, 2001; Roychowdhury, 2005; Afessa and Peters, 2006; Soubani and Uberti, 2007; Afessa et al., 2012). These occur throughout the different engraftment phases but BOS in particular, is more commonly seen late post-engraftment (Soubani and Uberti, 2007). Malignant relapse or secondary malignancy also complicate successful transplants (Copelan, 2006; Roziakova, 2011). Many times the non-infectious complications can manifest in conjunction with infectious complications (Metcalf et al., 1994; Roychowdhury, 2005). Additionally, infections are risk factors for development of some forms of non-infectious lung injury which may occur later, such as IPS (Versluys, 2010). Importantly, infections with bacterial, viral, and fungal pathogens can afflict both allogeneic and autologous HSCT recipients at multiple times post-transplant (Afessa and Peters, 2006; Lim, 2006).

Viral infections are common in HSCT patients. *Cytomegalovirus* (CMV) infections predominate in the late phase of engraftment and are associated with increased mortality (Boeckh, 2003, 2011; Kotloff et al., 2004). The advent of prophylactic and antiviral therapy usage has proven to be beneficial in limiting infection in HSCT patients; however, CMV can be particularly difficult to detect and treat (Boeckh, 2011). One autopsy study revealed that 65% of CMV infections were not diagnosed ante mortem, thus highlighting the frequency of missed detection (Chandrasekar, 1995). Thus, HSCT patients are highly susceptible to CMV as well as other herpesviruses (Soubani et al., 1996; Ljungman, 2002; Marr, 2012). Fungal pathogens are one of the major causes of significant morbidity and mortality in HSCT recipients (Marr et al., 2002). Prior to the use of azole-based anti-fungal prophylaxis, *Candida* spp. were the leading fungal pathogen following HSCT (Asano-Mori, 2010). However, following implementation of this anti-fungal prophylactic treatment, *Aspergillus* spp. have become the predominant fungal pathogen causing invasive fungal infections in the lung of both autologous and allogeneic HSCT populations (Bow, 2009; Asano-Mori, 2010). When considering bacterial infections, a shorter time to engraftment and the use of broad-spectrum antibiotics have improved the incidence of bacterial infections, specifically Gram-negative bacteria (Cruciani, 1996; Therriault, 2010). However, bacterial pneumonias remain a significant cause of mortality in this immunocompromised population (Therriault, 2010; Marr, 2012). *Pseudomonas aeruginosa* (a Gram-negative bacteria) is the most common pathogen isolated within 100 days post-engraftment and is associated with high recurrence rates due to increasing antibiotic resistance (Hakki et al., 2007). *Streptococcus pneumoniae* is the leading Gram-positive bacterial infection in HSCT recipients, and together with another Gram-positive agent, *S. aureus*, afflicts patients in the late post-engraftment phase and causes significant mortality (Chen et al., 2003; Marr, 2012).

## Animal Modeling

Murine bone marrow transplant (BMT) models for both syngeneic (genetically identical donor) and allogeneic (non-identical) transplantation offer the opportunity to study changes in the pulmonary immune system and microenvironment following transplantation. In these systems, donor cells are harvested from the femur and tibia of genetically inbred mice and introduced into lethally irradiated syngeneic or allogeneic mice to reconstitute ablated hematopoietic compartments. In order to force reconstitution of myeloid cells from hematopoietic donors, myeloablative conditioning is employed. This can be done via TBI or via chemotherapy regimens. One note of caution however, is that different strains of mice have differing susceptibilities to TBI dosage. For instance, in our laboratory we routinely use 13 Gy split dose of TBI to myeloablate C57Bl/6 mice and only 10 Gy for the same purpose in Balb/c mice. Addition of splenic T cells along with the donor bone marrow inoculum can hasten reconstitution in syngeneic transplants (Ojielo et al., 2003) and can be a source of alloreactive T cells in models of allogenic BMT hoping to study GvHD or graft versus leukemia responses (Reddy et al., 2005; Kato et al., 2010).

Studies comparing conditioning regimens for their ability to reconstitute AMs in recipient syngeneic BMT mice showed that both dual-chemotherapy regimens (4 days of 25 mg/kg busulfan followed by 2 days of 100 mg/kg cyclophosphamide) and 13 Gy TBI regimens induced greater than 50% reconstitution of AMs and spleen cells from donor stem cells by week 5 post-BMT (Hubbard et al., 2008). Thirteen Gray TBI, when compared to dual-chemotherapy, was the more effective conditioning regimen. With 13 Gy TBI conditioning, donor reconstitution, measured by the percentage of CD45.1 donor cells in CD45.2 recipient mice, was observed at 82 ± 2% in AMs and 95 ± 1% in spleen cells (Hubbard et al., 2008). The percentages of donor-derived AMs and spleen cells with dual-chemotherapy were 56 ± 6.2 and 72.3 ± 2.1% (Hubbard et al., 2008). Similar defects in host immune responses were noted in all of these myeloablative conditioning regimens regardless of whether T cells were included in the original inoculum (Hubbard et al., 2008).

## Defective Pulmonary Innate Immunity to Bacteria

### Bacterial infection model

Despite the use of antibiotic prophylaxis, infectious pulmonary complications continue to afflict both autologous and allogeneic HSCT recipients throughout the process of engraftment and well into the late post-engraftment phase (>100 days) (Chen et al., 2003; Marr, 2012). Although reported to predominantly manifest in the pre-engraftment and early post-engraftment phases, *P. aeruginosa-*driven pneumonia is also seen in some cases more than a year post-initial transplant (Chen et al., 2003). To understand how alterations following transplantation enhance susceptibility of HSCT patients to bacterial infections, we exposed syngeneic BMT mice to a sublethal dose of *P. aeruginosa* (PAO1 strain; 5 × 10^5^ CFU) (Ojielo et al., 2003; Ballinger et al., 2006a, 2008; Coomes et al., 2011) or *S. aureus* (USA300 stain; 7 × 10^7^ CFU) via intratracheal (i.t.) injection. Twenty-four hours post-infection, lungs and blood are collected to determine bacterial burden. Using this model, we found that syngeneic BMT mice are more susceptible to bacterial infections as they are unable to effectively clear *P. aeruginosa* (Ojielo et al., 2003; Ballinger et al., 2006a, 2008; Coomes et al., 2011; Domingo-Gonzalez et al., 2013) or *S. aureus* (Domingo-Gonzalez et al., 2013) from the lung and contain higher levels of bacterial dissemination compared to untransplanted, infected control mice. The remainder of this review will highlight the pathologic abnormalities which we have characterized in the innate immune cells using this model system. It should be noted that we utilized mice at week 5 post-BMT when hematopoietic reconstitution (including AMs) from donor HSCs was maximal (Hubbard et al., 2008). We assessed bacterial clearance 24 h post-infection and we used specific pathogen free mice; thus, there was little chance of co-infection. Using this model system, we have identified numerous BMT-induced changes that characterize the AMs and polymorphonuclear leukocytes (PMNs) that repopulate the host.

### Cytokine and eicosanoid dysregulation post-BMT

In humans, bacterial infection is commonly seen in the neutropenic phase due to a slow or incomplete engraftment, or the use of immunosuppressive therapy (Afessa and Peters, 2006; Coomes et al., 2011). However, in our syngeneic BMT model, there was prolonged susceptibility to bacterial infection even after full hematopoietic engraftment had occurred. These observations suggested that the environment of the lung post-BMT may be suppressing the function of the innate immune cells which had repopulated the alveolar space. These studies revealed that syngeneic BMT mice are unable to successfully induce TNFα or IFNγ in the lung following infection with *P. aeruginosa* (Ojielo et al., 2003). TNFα in particular, has been shown to be especially important in controlling a *P. aeruginosa* infection (Sapru et al., 1999; Chen et al., 2000). Balb/c mice are naturally resistant to *P. aeruginosa* and have been shown to produce higher levels of TNFα in the lung following infection while the susceptible C57Bl/6 strain produces significantly lower levels of TNFα post-infection (Gosselin, 1995; Morissette et al., 1995). Interestingly, *in vivo* treatment of Balb/c mice with an anti-murine TNFα monoclonal antibody reversed resistance to infection and the anti-TNFα-treated mice exhibited higher bacterial loads in the lung (Gosselin, 1995). Similarly, a separate study showed that TNFα-knockout mice suffered from significantly higher bacterial burden in the lungs following challenge with *P. aeruginosa* compared to their TNFα-sufficient wild-type controls (Yu, 2000). These data highlight the importance of TNFα in mediating clearance of *P. aeruginosa* and suggest that loss of the mediator post-BMT likely contributes to impaired clearance of this pathogen.

In addition to TNFα, IFNγ is also important for the activation of macrophages. IFNγ functions to prime macrophages to undergo classical activation but does not alone activate macrophages (Nathan, 1991; Mosser, 2003). It requires a second signal, provided by either exogenous TNFα or Toll-like receptor (TLR)-induced TNFα (57). However, as both IFNγ and TNFα were decreased in the alveolar space and TNFα production was decreased by AMs following BMT in response to i.t. challenge with *P. aeruginosa*, it is possible that the lack of activation signals for macrophages contribute to the inability to control and clear bacterial insult (Ojielo et al., 2003).

Eicosanoids derive from arachidonic acid and are lipid mediators produced by different cell types, including alveolar epithelial cells (AECs), AMs, and PMNs (Ballinger et al., 2006a, 2007; Folco and Murphy, 2006). Prostaglandins and leukotrienes are end-products of arachidonic acid metabolism via the cyclooxygenase (COX) and 5-lipoxygenase enzymatic pathways, respectively (Folco and Murphy, 2006). These eicosanoids have diverse effects on cells. Leukotrienes (LTB_4_ and cysteinyl leukotrienes or cys LTs) seem to have an overall pro-inflammatory effect on innate immune cells as they have been shown to promote bacterial phagocytosis and killing in both AMs and PMNs (Bailie, 1996; Mancuso et al., 1998; Mancuso, 2001; Serezani et al., 2005). Similarly, cys LTs have been shown to be involved in the induction of TNFα production by both AMs and recruited PMNs (Sayers, 1988; Ménard, 2000). Studies on the effects of prostaglandins, particularly prostaglandin E_2_ (PGE_2_), however, suggest they may negatively regulate innate immune responses (Aronoff et al., 2004). PGE_2_ signals through four distinct G protein-coupled seven transmembrane spanning E prostanoid (EP) receptors (Funk, 2001; Hubbard et al., 2001). The inhibitory effects of PGE_2_ have been attributed to binding of EP2 and EP4. Stimulation of the EP2 or EP4 receptors results in the induction of intracellular cyclic adenosine monophosphate (cAMP) and activation of the downstream cAMP targets: protein kinase A (PKA) and the exchange protein activated by cAMP (Epac-1) (Aronoff et al., 2004, 2005). Activation of PKA and/or Epac-1 has been shown to impair both AM and PMN function as well as inhibit the production of pro-inflammatory mediators, leukotrienes, and reactive oxygen species (He, 2001; Aronoff et al., 2005). Interestingly, PGE_2_ has also been shown to induce IL-10, an immunosuppressive cytokine (Harizi, 2002; MacKenzie et al., 2012).

Prostaglandin E_2_ has been reported to be elevated in the serum of autologous HSCT recipients (Cayeux et al., 1993). This increase in PGE_2_ was not specific to a particular conditioning regimen indicating that all HSCT patients are susceptible to overproduction of PGE_2_ (Cayeux et al., 1993). We have previously reported that PGE_2_ is elevated in the lungs of mice following syngenic BMT (Ballinger et al., 2006a). This overproduction was localized to AMs, recruited PMNs, and structural cells, whereas cys LTs are decreased in BMT AMs (Ballinger et al., 2006a). The increased production of PGE_2_ is likely to be systemic as BMT peritoneal lavage fluid also displayed higher PGE_2_ levels (Ballinger et al., 2006a). Although it is tempting to suspect PGE_2_-induced IL-10 expression may be responsible for the impaired immune environment post-BMT, IL-10 was decreased in the BMT lung following infectious stimuli indicating that the inhibitory effects of PGE_2_ were likely independent of the immunosuppressive effects of IL-10 (Ojielo et al., 2003). Interestingly, pharmacological inhibition of the COX pathway post-BMT limited PGE_2_ production and restored cysLT levels in AMs indicating that PGE_2_ negatively regulates cysLTs in the setting of BMT (Ballinger et al., 2006a). The importance of cysLTs in supporting the clearance and resolution of pulmonary infections post-BMT is observed in mice that received HSCs from granulocyte macrophage colony stimulating factor (GM-CSF)^−/−^ mice (Ballinger et al., 2008). GM-CSF^−/−^ BMT mice are defective in cysLT production post-infection and are more susceptible to *P. aeruginosa* infection.

Granulocyte macrophage colony stimulating factor is an important cytokine for regulating innate immune cells, particularly macrophages. Previous studies have shown that GM-CSF is required for effective clearance of *P. aeruginosa* even in untransplanted mice (Ballinger et al., 2006b). Untransplanted GM-CSF^−/−^ mice had increased bacterial burden compared to wild-type controls. However, the source and amount of GM-CSF is critical to determining host defense function post-BMT. AMs from wild-type syngeneic BMT mice show an overproduction of GM-CSF post-BMT. Interestingly, in human HSCT literature, elevated production of GM-CSF by AMs during the pancytopenia period was also noted (Whittle et al., 2001). While we initially thought excess AM-derived GM-CSF would be beneficial, this was not the case because the excess GM-CSF in AMs post-BMT drove upregulation of the EP2 receptor on AMs (Ballinger et al., 2008). The inhibitory effects of PGE_2_ signaling via upregulated EP2 in the wild-type BMT mice impaired bacterial phagocytosis and killing as well as cys LT and TNFα production. When GM-CSF^−/−^ HSCs were used to repopulate WT mice, host defense improved (Ballinger et al., 2008). Without AM-derived GM-CSF post-BMT, EP2 receptors were not elevated and mice showed improved host defense despite the fact that PGE_2_ levels were still elevated post-BMT. In the absence of EP2 elevations, the GM-CSF^−/−^ BMT mice retained production of cys LTs and TNFα. It is important to stress that improved host defense was only seen in the situation where GM-CSF production was blocked in hematopoietic cells post-transplant. When WT HSCs were transplanted into GM-CSF^−/−^ mice, host defense was once again impaired (Ballinger et al., 2008). These data highlight the importance of cross-talk between AECs and AMs post-BMT. AEC-derived GM-CSF is beneficial post-BMT whereas AM-derived GM-CSF is detrimental. In the human HSCT literature mentioned above, elevations of AM-derived GM-CSF in human allogenic HSCT recipients predicted development of later lung disease (Whittle et al., 2001). This complex homeostatic regulation of GM-CSF means blocking this cytokine needs to be targeted specifically to the AMs to be beneficial. Newer approaches to create microparticle delivery vesicles that are readily uptaken by phagocytes, but not structural cells may offer a way to deliver Ab or siRNA-based anti-GM-CSF therapies in the future (Jhunjhunwala and Little, 2011).

### Cellular alterations

#### Alveolar macrophages

##### Induction of PGE_2_ via cyclooxygenase-2 demethylation

Alveolar macrophages compose 95% of all leukocytes in the airspaces and thus, are the sentinel phagocytic cells in the lungs (Gordon and Read, 2002; Martin and Frevert, 2005). They have been implicated as critical innate immune cells for clearance of bacteria as depletion of AMs through clodronate liposomes resulted in impaired host defense against *P. aeruginosa* (Kooguchi et al., 1998). As AMs are key players in a productive innate immune response in the lungs, their function following transplantation is essential. However, data suggest that BMT AMs demonstrate altered cytokine and eicosanoid production which impair their innate immune function and predispose the host to infections post-BMT.

Prostaglandin E_2_ is significantly overproduced by BMT AMs (Ballinger et al., 2006a). The cause for elevated basal levels of PGE_2_ in the AMs post-BMT may be multifactorial. It is possible that differences in differentiation of AMs from donor-derived HSCs versus a resident AM stem cell may influence the amount of PGE_2_ observed after transplant (Tarling et al., 1987). However, studies indicate that this cannot fully explain the defect since mice receiving a lower dose of irradiation (8 Gy) contain a large percentage of host-derived AMs (∼36%) that also overproduce PGE_2_ post-transplant, albeit at approximately a threefold lower level than seen in AMs derived from donor HSCs (Hubbard et al., 2008). To further understand how PGE_2_ might be overproduced post-BMT, upstream regulators of PGE_2_ in the arachidonic acid metabolism cascade were analyzed. COX-2, the inducible form of COX, processes arachidonic acid to present to prostaglandin synthase enzymes to form prostaglandins (Funk, 2001). COX-2 expression is also increased following BMT (Domingo-Gonzalez, 2012). This increase in expression was due to significant hypomethylation of the COX-2 gene post-transplant. This demethylation induced expression of COX-2 and thus allowed increased PGE_2_ synthesis in the setting of BMT. The demethylation of the COX-2 gene was shown to be regulated at least in part via the actions of transforming growth factor beta (TGFβ), another cytokine that is elevated in the lungs post-BMT and is produced by the AECs (Domingo-Gonzalez, 2012). To determine the effects of TGFβ on COX-2 expression in BMT AMs, mice containing a dominant negative TGFβRII expressed under the CD11c promoter were used as donor mice (Domingo-Gonzalez, 2012). Thus, in these transplants, AMs would be insensitive to TGFβ signaling. These studies revealed that blocking TGFβ signaling restored methylation patterns within the COX-2 gene. Taken together, these studies indicate that elevations of TGFβ post-BMT induce COX-2 expression via demethylation of the gene (Domingo-Gonzalez, 2012). While not the subject of this review, it is interesting to note that elevations of TGFβ post-BMT also impair effector T cell responses and this alteration increases the susceptibility of BMT mice to herpesviral infections as well (Coomes et al., 2010, 2011).

Alveolar macrophages have been reported to exhibit decreased ability to phagocytose and kill bacteria following allogeneic HSCT (Winston et al., 1982). As AMs are important first responders to infection in the lung, their function is essential for preventing bacterial dissemination as well as promoting bacterial clearance by engulfment and removal of infectious pathogens, and also by releasing chemokines to stimulate the recruitment and activation of immune cells (Martin and Frevert, 2005). Murine BMT AMs display an impaired ability to phagocytose and kill *P. aeruginosa* (Ojielo et al., 2003; Ballinger et al., 2006a; Hubbard et al., 2010, 2011). This defect has been shown to be dependent on PGE_2_ signaling as pharmacological inhibition of the COX pathway through the use of indomethacin can restore a productive host defense against bacteria both *in vitro* and *in vivo* post-BMT. Furthermore, blocking the EP2 receptor (using AH 6809 a strong EP2 antagonist with weaker activity at the EP1 and DP1 receptors) in BMT AMs inhibits PGE_2_ signaling and restores phagocytosis of *P. aeruginosa* (Ballinger et al., 2006a). Thus, the next experiments discussed were undertaken to determine how PGE_2_ signaling inhibits AM phagocytosis and bacterial killing post-BMT.

##### Phosphatase and tensin homolog deleted on chromosome 10 (PTEN) elevations

Endocytosis of pathogens can occur in a number of ways with two major ways being through opsonized and non-opsonized phagocytosis. As bacterial uptake is impaired post-BMT, we sought to better understand the reasons for this. PTEN functions to dephosphorylate both protein and lipid targets, and has been traditionally known for its effects on PI3K/AKT signaling through dephosphorylation of phosphatidylinositol (Cutler, 2005; Copelan, 2006; Passweg, 2012)-triphosphate (PIP_3_) (Gunzl and Schabbauer, 2008). Previous studies have shown that PGE_2_-induced PTEN activity can inhibit FcγR-mediated phagocytosis and PI3K/AKT activation (Canetti et al., 2007). In the syngeneic BMT model, overproduction of PGE_2_ stimulates increased PTEN activity, resulting in decreased serum-opsonized phagocytosis (Hubbard et al., 2011). Using myeloid-specific PTEN conditional knockout (PTEN CKO) mice as BMT donors, recipient mice had improved, but not completely restored, host defense to *in vivo* bacterial challenge. This rescued response correlated with recovered serum-opsonized phagocytosis by AMs and improved killing of *P. aeruginosa* by both AMs and PMNs. Interestingly, non-opsonized phagocytosis in AMs was only partially recovered (Hubbard et al., 2011). This partial restoration may be due to persisted elevation of the IL-1 receptor-associated kinase-M (IRAK-M) post-BMT.

##### IRAK-M elevations

A member of the IRAK family of serine/threonine kinases, IRAK-M functions as a negative regulator of MyD88-dependent TLR signaling (Kobayashi, 2002). Our BMT model revealed that PGE_2_ can upregulate IRAK-M in AMs (Hubbard et al., 2010). Furthermore, BMT mice receiving genetically ablated IRAK-M^−/−^ donor HSCs were able to control and clear the bacterial infection similarly to untransplanted controls. This recovery also correlated with rescued TNFα, cys LTs, and AM function (including non-opsonized phagocytosis and killing) (Hubbard et al., 2010). Interestingly, these mice retained overproduction of PGE_2_ and overexpression of the inhibitory receptor, EP2. Thus, these data suggest that the inhibitory effects of PGE_2_ are dependent on IRAK-M and that IRAK-M is a critical downstream mediator of inhibitory PGE_2_ signaling (Hubbard et al., 2010). Taken together, these studies suggest that PGE_2_ signals through IRAK-M independently of PTEN and increased activity of IRAK-M and PTEN promote differential effects on opsonized and non-opsonized phagocytosis. Furthermore, the PTEN CKO studies highlight the importance of non-opsonized phagocytic responses in the lung following transplant, as complete recovery of serum-opsonized phagocytosis and bacterial killing did not completely abrogate *in vivo* susceptibility measured 24 h post-infection (Hubbard et al., 2011).

##### Scavenger receptor alterations

Non-opsonized phagocytosis is primarily mediated by scavenger receptors (SRs), specifically Class A SRs (Palecanda and Kobzik, 2001). Within this family of SRs are SR-AI and II, macrophage receptor with a collagenous structure (MARCO), SR with C-type lectin (SRCL), and SR receptor A5 (SCARA5) (Peiser et al., 2002). SR-AI and II are splice variants, and together with MARCO, have been associated with mediating non-opsonized phagocytosis in macrophages (Peiser et al., 2002). Their importance in bacterial clearance is highlighted by the susceptibility of individual MARCO^−/−^ and SR-AI/II^−/−^ mice to *S. pneumoniae* (Arredouani et al., 2004, 2006). Defective phagocytosis of *S. pneumoniae* in SR-AI/II^−/−^ mice has also been reported (Arredouani et al., 2006). Interestingly, the impaired phagocytosis of non-opsonized *P. aeruginosa* in our BMT model correlated with aberrant SR profiles (decreased MARCO and increased SR-AI/II expression) (Domingo-Gonzalez et al., 2013). Treatment of untransplanted AMs with soluble MARCO, inhibited phagocytosis similarly to pan class A SR inhibitors and was comparable to the level of phagocytosis noted against *P. aeruginosa* in wild-type BMT AMs. In contrast, SR-AI/II overexpression explained the surprising enhancement of *S. aureus* phagocytosis by BMT AMs (Domingo-Gonzalez et al., 2013).

The differences in SR profiles observed following transplant can be attributed to PGE_2_. Exogenous treatment of naïve AMs with PGE_2_ decreased MARCO and increased SR-AI/II considerably (Domingo-Gonzalez et al., 2013). The mechanism(s) explaining how PGE_2_ is able to regulate SR expression is not fully characterized. However, our studies revealed that PGE_2_ can inhibit expression of microRNA-155 (miR-155) (Domingo-Gonzalez et al., 2013). MicroRNAs are small non-coding RNAs that typically target 3′untranslated regions (3′UTR) and can inhibit or stabilize mRNA expression or in many cases inhibit mRNA translation (He and Hannon, 2004; Chen and Rajewsky, 2007). miR-155 has been generally shown to play a role in promoting an inflammatory response as it has been shown to stabilize TNFα mRNA and thus promote expression (Bala et al., 1436). In the context of transplant, miR-155 expression is decreased by PGE_2_ (Domingo-Gonzalez et al., 2013). Interestingly, the 3′UTR of SR-AI/II contains a putative miR-155 target sequence (TargetScan). Blocking miR-155 can induce expression of SR-AI/II and result in enhanced phagocytosis of *S. aureus* (Domingo-Gonzalez et al., 2013). These results suggest that by negatively regulating an inhibitor of SR-AI/II, PGE_2_ can indirectly induce expression of this SR. The mechanism for how PGE_2_ reduces MARCO expression is unknown at present, but does not involve miR-155. Importantly, despite differences in levels of phagocytosis of *P. aeruginosa* and *S. aureus*, a defect in killing remains in BMT AMs which ultimately confers susceptibility to both bacterial infections (Domingo-Gonzalez et al., 2013). As mentioned above, this killing defect may relate to both IRAK-M elevation and PTEN activation post-BMT. Loss of miR-155 in response to PGE_2_ signaling may also serve to destabilize TNFα mRNA post-BMT, and this is also likely to impair bacterial killing.

#### Lung epithelial cells

Alveolar epithelial cells are the structural cells lining the alveolus. Type II AECs are important secretors of pulmonary surfactant, are progenitors to type I AECs which line the majority of the lung, and act as immunomodulators via interactions with immune cells. Exposure to ionizing radiation induces damage to the alveolar epithelial barrier causing changes in gene expression, particularly cytokine expression (Rubin et al., 1995). We have previously shown that upon transplantation, AECs from BMT mice are negatively affected by the transplant process. Specifically, AECs overproduce PGE_2_ as well as TGFβ which as discussed above inhibits AM function (Ballinger et al., 2006a; Coomes et al., 2010; Domingo-Gonzalez, 2012). Furthermore, AECs are defective in the synthesis of GM-CSF (Ballinger et al., 2006b, 2008) which as discussed above is important for modulating AM function. Another important defect that has been described post-BMT is the loss of surfactant protein A (SPA) which is predominantly synthesized by AECs (Yang, 2000; Wright, 2005). As SPA can serve as a collectin (Wright, 2005) which can bind pathogens to help facilitate phagocytosis via the SR-AI/II receptor (Kuronuma, 2004), loss of SPA post-BMT may be another reason why host defense is impaired in the setting of HSCT. The fact that SPA can bind to SR-AI/II (the receptor that is retained on BMT AMs) may suggest that exogenous SPA therapy could prove beneficial post-HSCT.

#### Polymorphonuclear leukocytes

A major role for PMNs lies in their ability to be recruited to the lung upon inflammation or infection and assist in the killing and ultimate clearance of pathogens (Wang, 2004; Koh, 2009). Several studies have looked at the function of human PMNs post-autologous HSCT (reviewed in Ramaprasad et al., 2010). These human studies noted decreased respiratory burst, decreased phagocytosis of *C. albicans* and decreased killing of *S. aureus* (Gadish et al., 1991). Interestingly the role of GM-CSF in restoring PMN function is mixed (Peters et al., 1988; Gadish et al., 1991; Wiltschke et al., 1995).

In the context of murine syngeneic BMT, PMNs were shown to have intact phagocytic ability but were defective in their ability to kill the engulfed bacteria (Ballinger et al., 2006a). Genetic ablation of GM-CSF in donor marrow improved, but did not fully restore, PMN killing of *P. aeruginosa* (Ballinger et al., 2008).

PTEN-deficient neutrophils have been reported to exhibit enhanced host immune responses (Subramanian et al., 2007; Li et al., 2009). Previous studies had shown that alleviating the repressive effect of PTEN on neutrophils, augmented neutrophil chemotaxis (Subramanian et al., 2007). Similar to these studies, our PTEN CKO BMT mice had elevated basal levels of PMNs in the alveolar space compared to untransplanted wild-type controls and recovered their ability to kill intracellular bacteria (Hubbard et al., 2011). However, despite a rescued PMN phenotype, PTEN CKO BMT mice were still susceptible to acute *P. aeruginosa* infection, highlighting the importance AM function, specifically the need for non-opsonized phagocytosis, post-BMT.

Together with AMs, the impaired function of PMNs can also be attributed to elevated PGE_2_, as pharmacological inhibition of PGE_2_ synthesis completely resolved their defect (Ballinger et al., 2006a). Unlike BMT AMs, PMNs do not exhibit a phagocytic defect and IRAK-M is not elevated in recruited PMNs. Thus, it is likely that PGE_2_ is mediating impaired killing through PTEN, as we have previously shown that PGE_2_ can induce PTEN activity (Hubbard et al., 2011).

## Conclusion

The cumulative data from our studies of murine syngeneic BMT is summarized in Table 1 and suggests that the susceptibility to pulmonary infections following HSCT is related to the prolonged impairment of immune function, particularly of AMs and PMNs, as well as cytokine alterations by AECs. The master innate immune inhibitory mediator post-BMT appears to be PGE_2_. Defects of AM phagocytosis and killing as well as defects in PMN killing limit the ability of the host to fight off bacterial infections post-HSCT. Blockade of PGE_2_ signaling or production can restore innate immune function. Work using this BMT mouse model provides critical insight into the causes of defective pulmonary immunity observed in the setting of transplantation and in the absence of immunosuppressive therapy or the confounding effects of GvHD.

**Table 1:** **Summary of observed cellular alterations following syn-BMT**.

	AECs	AMs	PMNs
Cytokines (baseline and post-infection)	↑TGFβ, PGE_2_, ↓GM-CSF	↑GM-CSF, PGE_2_, ↓cysLTs, TNFα	↑PGE_2_
Function		↓Phagocytosis, bacterial killing	↓Bacterial killing
Intracellular signaling		↑PTEN, IRAK-M	Loss of PTEN improves function, IRAK-M levels do not change
miRNA		↓miR-155	
Scavenger receptors		↑SR-AI/II, ↓MARCO	

**Figure 1:**
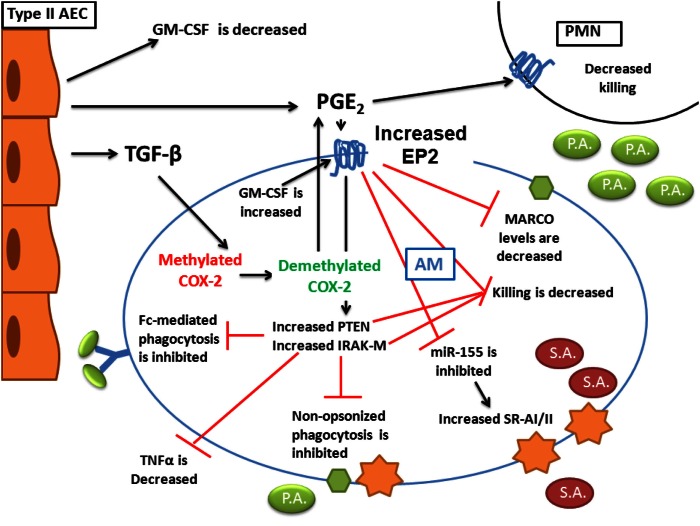
**Alterations in innate immunity post-BMT**. AEC production of TGFβ and PGE_2_ are increased following transplant and both affect AM function. AEC production of GM-CSF is reduced. TGFβ signaling in AMs helps demethylate the COX-2 promoter to increase AM-derived PGE_2_ secretion. Overproduction of GM-CSF in AMs post-BMT leads to elevation of the EP2 receptor. In response to PGE_2_ stimulation of increased EP2 receptors, AM phagocytosis of both opsonized and unopsonized pathogens is impaired. This impaired phagocytosis is related to a loss of MARCO expression as well as an upregulation of IRAK-M. Despite the fact that PGE_2_ reduces miR-155 which leads to increased SR-AI/II expression, bacterial killing in both AMs and PMNs is impaired. This is related to activation of PTEN as well as increased IRAK-M. Not shown on the diagram is the observation that TNFα expression in AMs is also lost as a result of PGE_2_ signaling. We have demonstrated that these alterations ultimately lead to impaired host defense against both Gram-negative *P. aeruginosa* as well as Gram-positive *S. aureus*.

Changes that characterize BMT AMs are diverse. Figure 1 provides a schematic overview of the changes that occur in AMs following transplant. It is interesting that most of the changes that occur can be attributed to eicosanoid dysregulation and the enhanced signaling of PGE_2_ through overexpression of one of its receptors, EP2. Signaling via EP2 regulates downstream targets; IRAK-M is increased and PTEN is activated post-BMT. The end result of these alterations is the impairment of AM and PMN function. The use of general COX inhibitors (e.g., indomethacin or aspirin) or COX-2-specific inhibitors (e.g., NS-398) may prove beneficial to HSCT patients. More recently, the development of PF-044148948, the first specific EP2 antagonist developed by Pfizer (Wang, 2004) may offer the exciting advantage of blocking the inhibitory effects of PGE_2_ signaling on AMs without shutting down all prostaglandin production or effecting other EP receptor signaling. Such a therapeutic may offer protection to HSCT patients suffering from antibiotic resistant bacterial infections. Finally, murine studies need to be confirmed in human HSCT AMs. If elevations in COX-2 and EP2 signaling as well as loss of SPA noted in murine studies can be verified in humans post-HSCT, the next step will be to design clinical trials to test therapies targeted at these transplant-induced alterations.

## Conflict of Interest Statement

The authors declare that the research was conducted in the absence of any commercial or financial relationships that could be construed as a potential conflict of interest.

## References

[B1] Afessa B. (2001). Bronchiolitis obliterans and other late onset non-infectious pulmonary complications in hematopoietic stem cell transplantation. Bone Marrow Transplant. 28, 425–434 10.1038/sj.bmt.1703142 11593314

[B2] Afessa B. Abdulai R. M. Kremers W. K. Hogan W. J. Litzow M. R. Peters S. G. (2012). Risk factors and outcome of pulmonary complications after autologous hematopoietic stem cell transplanthematopoietic stem cell transplant complications. Chest 141, 442–450 10.1378/chest.10-2889 21778261

[B3] Afessa B. Peters S. G. (2006). Major complications following hematopoietic stem cell transplantation. Semin. Respir. Crit. Care Med. 27, 297–309 10.1055/s-2006-945530 16791762

[B4] Aronoff D. M. Canetti C. Peters-Golden M. (2004). Prostaglandin E2 inhibits alveolar macrophage phagocytosis through an E-prostanoid 2 receptor-mediated increase in intracellular cyclic AMP. J. Immunol. 173, 559–565 15210817 10.4049/jimmunol.173.1.559

[B5] Aronoff D. M. Canetti C. Serezani C. H. Luo M. Peters-Golden M. (2005). Cutting edge: macrophage inhibition by cyclic AMP (cAMP): differential roles of protein kinase A and exchange protein directly activated by cAMP-1. J. Immunol. 174, 595–599 15634874 10.4049/jimmunol.174.2.595

[B6] Arredouani M. Yang Z. Ning Y. Qin G. Soininen R. Tryggvason K. (2004). The scavenger receptor MARCO is required for lung defense against pneumococcal pneumonia and inhaled particles. J. Exp. Med. 200, 267–272 10.1084/jem.20040731 15263032 PMC2212010

[B7] Arredouani M. S. Yang Z. Imrich A. Ning Y. Qin G. Kobzik L. (2006). The macrophage scavenger receptor SR-AI/II and lung defense against pneumococci and particles. Am. J. Respir. Cell Mol. Biol. 35, 474–478 10.1165/rcmb.2006-0128OC 16675784 PMC2643266

[B8] Asano-Mori Y. (2010). Fungal infections after hematopoietic stem cell transplantation. Int. J. Hematol. 91, 576–587 10.1007/s12185-010-0574-0 20432074

[B9] Aschan J. (2007). Risk assessment in haematopoietic stem cell transplantation: conditioning. Best Pract. Res. Clin. Haematol. 20, 295–310 10.1016/j.beha.2006.09.004 17448963

[B10] Bailie M. B. (1996). Leukotriene-deficient mice manifest enhanced lethality from *Klebsiella* pneumonia in association with decreased alveolar macrophage phagocytic and bactericidal activities. J. Immunol. 157, 5221–5224 8955165

[B11] Bala S. Marcos M. Kodys K. Csak T. Catalano D. Mandrekar P. (1436). Up-regulation of microRNA-155 in macrophages contributes to increased tumor necrosis factor {alpha} (TNF{alpha}) production via increased mRNA half-life in alcoholic liver disease. J. Biol. Chem. 286, 1436–1444 10.1074/jbc.M110.145870 21062749 PMC3020752

[B12] Ballinger M. N. Aronoff D. M. McMillan T. R. Cooke K. R. Okiewicz K. Toews G. B. (2006a). Critical role of prostaglandin E2 overproduction in impaired pulmonary host response following bone marrow transplantation. J. Immunol. 177, 5499–5508 17015736 10.4049/jimmunol.177.8.5499

[B13] Ballinger M. N. Paine R. III Serezani C. H. Aronoff D. M. Choi E. S. Standiford T. J. (2006b). Role of granulocyte macrophage colony-stimulating factor during gram-negative lung infection with *Pseudomonas aeruginosa*. Am. J. Respir. Cell Mol. Biol. 34, 766–774 10.1165/rcmb.2005-0246OC 16474098 PMC2644237

[B14] Ballinger M. N. Hubbard L. L. McMillan T. R. Toews G. B. Peters-Golden M. Paine R. III (2008). Paradoxical role of alveolar macrophage-derived granulocyte-macrophage colony-stimulating factor in pulmonary host defense post-bone marrow transplantation. Am. J. Physiol. Lung Cell Mol. Physiol. 295, L114–L122 10.1152/ajplung.00309.2007 18456799 PMC2494794

[B15] Ballinger M. N. McMillan T. R. Moore B. B. (2007). Eicosanoid regulation of pulmonary innate immunity post-hematopoietic stem cell transplantation. Arch. Immunol. Ther. Exp. 55, 1–12 10.1007/s00005-007-0001-2 17221337 PMC3313470

[B16] Boeckh M. (2003). *Cytomegalovirus* in hematopoietic stem cell transplant recipients: current status, known challenges, and future strategies. Biol. Blood Marrow Transplant. 9, 543–558 10.1016/S1083-8791(03)00287-8 14506657

[B17] Boeckh M. (2011). Complications, diagnosis, management, and prevention of CMV infections: current and future. Hematology Am. Soc. Hematol. Educ. Program 2011, 305–309 10.1182/asheducation-2011.1.305 22160050

[B18] Bow E. J. (2009). Invasive fungal infection in haematopoietic stem cell transplant recipients: epidemiology from the transplant physician’s viewpoint. Mycopathologia 168, 283–297 10.1007/s11046-009-9196-6 19343534

[B19] Canetti C. Serezani C. H. Atrasz R. G. White E. S. Aronoff D. M. Peters-Golden M. (2007). Activation of phosphatase and tensin homolog on chromosome 10 mediates the inhibition of FcgammaR phagocytosis by prostaglandin E2 in alveolar macrophages. J. Immunol. 179, 8350–8356 18056380 10.4049/jimmunol.179.12.8350

[B20] Cayeux S. J. Beverly P. C. L. Schulz R. Dorken B. (1993). Elevated plasma prostaglandin E2 levels found in 14 patients undergoing autologous or stem cell transplantation. Bone Marrow Transplant. 12, 603–608 7907905

[B21] Chandrasekar P. H. (1995). Autopsy-identified infections among bone marrow transplant recipients: a clinico-pathologic study of 56 patients. Bone Marrow Transplantation Team. Bone Marrow Transplant. 16, 675–681 8547865

[B22] Chen C.-S. Boeckh M. Seidel K. Clark J. G. Kansu E. Madtes D. K. (2003). Incidence, risk factors, and mortality from pneumonia developing late after hematopoietic stem cell transplantation. Bone Marrow Transplant. 32, 515–522 10.1038/sj.bmt.1704162 12942099

[B23] Chen G. H. Reddy R. C. Newstead M. W. Tateda K. Kyasapura B. L. Standiford T. J. (2000). Intrapulmonary TNF gene therapy reverses sepsis-induced suppression of lung antibacterial host defense. J. Immunol. 165, 6496–6503 11086090 10.4049/jimmunol.165.11.6496

[B24] Chen K. Rajewsky N. (2007). The evolution of gene regulation by transcription factors and microRNAs. Nat. Rev. Genet. 8, 93–103 10.1038/nrg1990 17230196

[B25] Coomes S. M. Hubbard L. L. Moore B. B. (2011). Impaired pulmonary immunity post-bone marrow transplant. Immunol. Res. 50, 78–86 10.1007/s12026-010-8200-z 21170739 PMC3310400

[B26] Coomes S. M. Wilke C. A. Moore T. A. Moore B. B. (2010). Induction of TGF-beta 1, not regulatory T cells, impairs antiviral immunity in the lung following bone marrow transplant. J. Immunol. 184, 5130–5140 10.4049/jimmunol.0901871 20348421 PMC3314499

[B27] Copelan E. A. (2006). Hematopoietic stem-cell transplantation. N. Engl. J. Med. 354, 1813–1826 10.1056/NEJMra052638 16641398

[B28] Cordonnier C. (1986). Pulmonary complications occurring after allogeneic bone marrow transplantation. A study of 130 consecutive transplanted patients. Cancer 58, 1047–1054 10.1002/1097-0142(19860901)58:5<1047::AIDCNCR2820580512>3.0.CO;2-Y 3524798

[B29] Cruciani M. (1996). Prophylaxis with fluoroquinolones for bacterial infections in neutropenic patients: a meta-analysis. Clin. Infect. Dis. 23, 795–805 10.1093/clinids/23.4.795 8909847

[B30] Cutler C. (2005). An overview of hematopoietic stem cell transplantation. Clin. Chest Med. 26, 517–527 10.1016/j.ccm.2005.06.016 16263393

[B31] Domingo-Gonzalez R. (2012). COX-2 expression is upregulated by DNA hypomethylation after hematopoietic stem cell transplantation. J. Immunol. 189, 4528–4536 10.4049/jimmunol.1201116 23008450 PMC3478470

[B32] Domingo-Gonzalez R. Katz S. Serezani C. Moore T. LeVine A. Moore B. (2013). PGE_2_-induced changes in alveolar macrophage scavenger receptor profiles differentially alter phagocytosis of *P. aeruginosa and S. aureus* post-bone marrow transplant. J. Immunol. 10.4049/jimmunol.1203274 (in press) 23630358 PMC3660503

[B33] Folco G. Murphy R. C. (2006). Eicosanoid transcellular biosynthesis: from cell-cell interactions to in vivo tissue responses. Pharmacol. Rev. 58, 375–388 10.1124/pr.58.3.8 16968946

[B34] Funk C. D. (2001). Prostaglandins and leukotrienes: advances in eicosanoid biology. Science 294, 1871–1875 10.1126/science.294.5548.1871 11729303

[B35] Gadish M. Kletter Y. Flidel O. Nagler A. Slavin S. Fabian I. (1991). Effects of recombinant human granulocyte and granulocyte-macrophage colony-stimulating factors on neutrophil function following autologous bone marrow transplantation. Leuk. Res. 15, 1175–1182 10.1016/0145-2126(91)90187-X 1722549

[B36] Giralt S. (2005). Reduced-intensity conditioning regimens for hematologic malignancies: what have we learned over the last 10 years? Hematology Am. Soc. Hematol. Educ. Program 2005, 384–389 10.1182/asheducation-2005.1.384 16304408

[B37] Gordon S. B. Read R. C. (2002). Macrophage defences against respiratory tract infections. Br. Med. Bull. 61, 45–61 10.1093/bmb/61.1.45 11997298

[B38] Gosselin D. (1995). Role of tumor necrosis factor alpha in innate resistance to mouse pulmonary infection with *Pseudomonas aeruginosa*. Infect. Immun. 63, 3272–3278 7642255 10.1128/iai.63.9.3272-3278.1995 PMC173451

[B39] Gratwohl A. Baldomero H. Aljurf M. Pasquini M. C. Bouzas L. F. Yoshimi A. (2010). Hematopoietic stem cell transplantation: a global perspective. JAMA 303, 1617–1624 10.1001/jama.2010.491 20424252 PMC3219875

[B40] Griese M. (2000). Pulmonary complications after bone marrow transplantation in children: twenty-four years of experience in a single pediatric center. Pediatr. Pulmonol. 30, 393–401 10.1002/1099-0496(200011)30:5<393::AIDPPUL5>3.0.CO;2-W 11064430

[B41] Gunzl P. Schabbauer G. (2008). Recent advances in the genetic analysis of PTEN and PI3K innate immune properties. Immunobiology 213, 759–765 10.1016/j.imbio.2008.07.028 18926291

[B42] Hakki M. Limaye A. P. Kim H. W. Kirby K. A. Corey L. Boeckh M. (2007). Invasive *Pseudomonas aeruginosa* infections: high rate of recurrence and mortality after hematopoietic cell transplantation. Bone Marrow Transplant. 39, 687–693 10.1038/sj.bmt.1705653 17401395

[B43] Harizi H. (2002). Cyclooxygenase-2-issued prostaglandin e(2) enhances the production of endogenous IL-10, which down-regulates dendritic cell functions. J. Immunol. 168, 2255–2263 11859113 10.4049/jimmunol.168.5.2255

[B44] He L. Hannon G. J. (2004). MicroRNAs: small RNAs with a big role in gene regulation. Nat. Rev. Genet. 5, 522–531 10.1038/nrg1415 15211354

[B45] He L. K. (2001). The expression of cyclooxygenase and the production of prostaglandin E2 in neutrophils after burn injury and infection. J. Burn Care Rehabil. 22, 58–64 10.1097/00004630-200101000-00012 11227686

[B46] Hubbard L. Wilke C. White E. Moore B. P. T. E. N. (2011). Limits alveolar macrophage function against *Pseudomonas aeruginosa* following bone marrow transplantation. Am. J. Respir. Cell Mol. Biol. 45, 1050–1058 10.1165/rcmb.2011-0079OC 21527775 PMC3361361

[B47] Hubbard L. L. Ballinger M. N. Thomas P. E. Wilke C. A. Standiford T. J. Kobayashi K. S. (2010). A role for IL-1 receptor-associated kinase-M in prostaglandin E2-induced immunosuppression post-bone marrow transplantation. J. Immunol. 184, 6299–6308 10.4049/jimmunol.0902828 20439918 PMC4040537

[B48] Hubbard L. L. Ballinger M. N. Wilke C. A. Moore B. B. (2008). Comparison of conditioning regimens for alveolar macrophage reconstitution and innate immune function post bone marrow transplant. Exp. Lung Res. 34, 263–275 10.1080/01902140802022518 18465404 PMC3309395

[B49] Hubbard N. E. Lee S. Lim D. Erickson K. L. (2001). Differential mRNA expression of prostaglandin receptor subtypes in macrophage activation. Prostaglandins Leukot. Essent. Fatty Acids 65, 287–294 10.1054/plef.2001.0327 11993722

[B50] Jhunjhunwala S. Little S. R. (2011). Microparticulate systems for targeted drug delivery to phagocytes. Cell 10, 2047–2048 10.4161/cc.10.13.15713 PMC3154357 21623160

[B51] Kato K. Cui S. Kuick R. Mineishi S. Hexner E. Ferrara J. L. (2010). Identification of stem cell transcriptional programs normally expressed in embryonic and neural stem cells in alloreactive CD8+ T cells mediating graft-versus-host disease. Biol. Blood Marrow Transplant. 16, 751–771 _ANY_ 20116439 10.1016/j.bbmt.2010.01.012 PMC2913321

[B52] Kim S. H. Kee S. Y. Lee D. G. Choi S. M. Park S. H. Kwon J. C. (2013). Infectious complications following allogeneic stem cell transplantation: reduced-intensity vs. myeloablative conditioning regimens. Transpl. Infect. Dis. 15, 49–59 10.1111/tid.12003 22998745

[B53] Kobayashi K. (2002). IRAK-M is a negative regulator of Toll-like receptor signaling. Cell 110, 191–202 10.1016/S0092-8674(02)00827-9 12150927

[B54] Koh A. Y. (2009). Inescapable need for neutrophils as mediators of cellular innate immunity to acute *Pseudomonas aeruginosa* pneumonia. Infect. Immun. 77, 5300–5310 10.1128/IAI.00501-09 19805527 PMC2786465

[B55] Kolb H.-J. (2008). Graft-versus-leukemia effects of transplantation and donor lymphocytes. Blood 112, 4371–4383 10.1182/blood-2008-03-077974 19029455

[B56] Kooguchi K. Hashimoto S. Kobayashi A. Kitamura Y. Kudoh I. Wiener-Kronish J. (1998). Role of alveolar macrophages in initiation and regulation of inflammation in *Pseudomonas aeruginosa* pneumonia. Infect. Immun. 66, 3164–3169 9632581 10.1128/iai.66.7.3164-3169.1998 PMC108328

[B57] Körbling M. Anderlini P. (2001). Peripheral blood stem cell versus bone marrow allotransplantation: does the source of hematopoietic stem cells matter? Blood 98, 2900–2908 10.1182/blood.V98.10.2900 11698269

[B58] Kotloff R. Ahya V. Crawford S. W. (2004). Pulmonary complications of solid organ and hematopoietic stem cell transplantation. Am. J. Respir. Crit. Care Med. 170, 22–48 10.1164/rccm.200309-1322SO 15070821

[B59] Kuronuma K. (2004). Pulmonary surfactant protein A augments the phagocytosis of *Streptococcus pneumoniae* by alveolar macrophages through a casein kinase 2-dependent increase of cell surface localization of scavenger receptor A. J. Biol. Chem. 279, 21421–21430 10.1074/jbc.M312490200 14993215

[B60] Li Y. Jia Y. Pichavant M. Loison F. Sarraj B. Kasorn A. (2009). Targeted deletion of tumor suppressor PTEN augments neutrophil function and enhances host defense in neutropenia-associated pneumonia. Blood 113, 4930–4941 10.1182/blood-2008-06-161414 19286998 PMC2686143

[B61] Lim D. H. (2006). Pulmonary complications after hematopoietic stem cell transplantation. J. Korean Med. Sci. 21, 406–411 10.3346/jkms.2006.21.3.406 16778380 PMC2729942

[B62] Little M.-T. Storb R. (2002). History of haematopoietic stem-cell transplantation. Nat. Rev. Cancer 2, 231–238 10.1038/nrc748 11990860

[B63] Ljungman P. (2002). Prevention and treatment of viral infections in stem cell transplant recipients. Br. J. Haematol. 118, 44–57 10.1046/j.1365-2141.2002.03515.x 12100127

[B64] Lorenz E. (1951). Modification of irradiation injury in mice and guinea pigs by bone marrow injections. J. Natl. Cancer Inst. 12, 197–201 14874130

[B65] MacKenzie K. F. Clark K. Naqvi S. McGuire V. A. Nöehren G. Kristariyanto Y. (2012). PGE2 induces macrophage IL-10 production and a regulatory-like phenotype via a protein kinase A–SIK–CRTC3 pathway. J. Immunol. 190, 565–577 10.4049/jimmunol.1202462 23241891 PMC3620524

[B66] Mancuso P. (2001). Leukotriene B4 augments neutrophil phagocytosis of *Klebsiella pneumoniae*. Infect. Immun. 69, 2011–2016 10.1128/IAI.69.4.2011-2016.2001 11254552 PMC98124

[B67] Mancuso P. Standiford T. J. Marshall T. Peters-Golden M. (1998). 5-Lipoxygenase reaction products modulate alveolar macrophage phagocytosis of *Klebsiella pneumoniae*. Infect. Immun. 66, 5140–5146 9784515 10.1128/iai.66.11.5140-5146.1998 PMC108641

[B68] Marr K. Carter R. Boeckh M. Martin P. Corey L. (2002). Invasive aspergillosis in allogeneic stem cell transplant recipients: changes in epidemiology and risk factors. Blood 100, 4358–4366 10.1182/blood-2002-05-1496 12393425

[B69] Marr K. A. (2012). Delayed opportunistic infections in hematopoietic stem cell transplantation patients: a surmountable challenge. Hematology Am. Soc. Hematol. Educ. Program 2012, 265–270 10.1182/asheducation-2012.1.265 23233590 PMC4696052

[B70] Martin T. R. Frevert C. W. (2005). Innate immunity in the lungs. Proc. Am. Thorac. Soc. 2, 403–411 10.1513/pats.200508-090JS 16322590 PMC2713330

[B71] Ménard G. (2000). Priming of alveolar macrophages by leukotriene D(4): potentiation of inflammation. Am. J. Respir. Cell Mol. Biol. 23, 572–577 10.1165/ajrcmb.23.4.4152 11017925

[B72] Metcalf J. P. Rennard S. I. Reed E. C. Haire W. D. Sisson J. H. Walter T. (1994). Corticosteroids as adjunctive therapy for diffuse alveolar hemorrhage associated with bone marrow transplantation. Am. J. Med. 96, 327–334 10.1016/0002-9343(94)90062-0 8166151

[B73] Morissette C. Skamene E. Gervais F. (1995). Endobronchial inflammation following *Pseudomonas aeruginosa* infection in resistant and susceptible strains of mice. Infect. Immun. 63, 1718–1724 7729877 10.1128/iai.63.5.1718-1724.1995 PMC173215

[B74] Mosser D. M. (2003). The many faces of macrophage activation. J. Leukoc. Biol. 73, 209–212 10.1189/jlb.0602325 12554797

[B75] Nathan C. (1991). Mechanisms and modulation of macrophage activation. Behring Inst. Mitt. 88, 200–207 2049039

[B76] Ojielo C. Cooke K. R. Mancuso P. Standiford T. J. Olkiewicz K. M. Cloutheir S. (2003). Defective phagocytosis and clearance of *Pseudomonas aeruginosa* in the lung following bone marrow transplantation. J. Immunol. 171, 4416–4424 14530368 10.4049/jimmunol.171.8.4416

[B77] Palecanda A. Kobzik L. (2001). Receptors for unopsonized particles: the role of alveolar macrophage scavenger receptors. Curr. Mol. Med. 1, 589–595 10.2174/1566524013363384 11899233

[B78] Passweg J. R. (2012). Hematopoietic stem cell transplantation: a review and recommendations for follow-up care for the general practitioner. Swiss. Med. Wkly. 142, w13696 10.4414/smw.2012.13696 23135685

[B79] Peffault de Latour R. Rocha V. Socie G. (2013). Cord blood transplantation in aplastic anemia. Bone Marrow Transplant. 48, 201–202 10.1038/bmt.2012.252 23292234

[B80] Peiser L. Mukhopadhyay S. Gordon S. (2002). Scavenger receptors in innate immunity. Curr. Opin. Immunol. 14, 123–128 10.1016/S0952-7915(01)00307-7 11790542

[B81] Peters W. P. Stuart A. Affronti M. L. Kim C. S. Coleman R. E. (1988). Neutrophil migration is defective during recombinant human granulocyte-macrophage colony-stimulating factor infusion after autologous bone marrow transplantation in humans. Blood 72, 1310–1315 3048440

[B82] Ramaprasad C. Pouch S. Pitrak D. L. (2010). Neutrophil function after bone marrow and hematopoietic stem cell transplant. Leuk. Lymphoma 51, 756–767 10.3109/10428191003695678 20350278

[B83] Reddy P. Maeda Y. Liu C. Krijanovski O. I. Korngold R. Ferrara J. L. (2005). A crucial role for antigen-presenting cells and alloantigen expression in graft-versus-leukemia responses. Nat. Med. 11, 1244–1249 10.1038/nm1309 16227991

[B84] Roychowdhury M. (2005). Pulmonary complications after bone marrow transplantation: an autopsy study from a large transplantation center. Arch. Pathol. Lab. Med. 129, 366–371 15737032 10.5858/2005-129-366-PCABMT

[B85] Roziakova L. (2011). Secondary malignancies after hematopoietic stem cell transplantation. Neoplasma 58, 1–8 10.4149/neo_2011_01_1 21067259

[B86] Rubin P. Johnston C. J. Williams J. P. McDonald S. Finkelstein J. N. (1995). A perpetual cascade of cytokines postirradiation leads to pulmonary fibrosis. Int. J. Radiat. Oncol. Biol. Phys. 33, 99–109 10.1016/0360-3016(95)00095-G 7642437

[B87] Sapru K. Stotland P. K. Stevenson M. M. (1999). Quantitative and qualitative differences in bronchoalveolar inflammatory cells in *Pseudomonas aeruginosa*-resistant and -susceptible mice. Clin. Exp. Immunol. 115, 103–109 10.1046/j.1365-2249.1999.00762.x 9933427 PMC1905184

[B88] Sayers T. J. (1988). Effect of cytokines on polymorphonuclear neutrophil infiltration in the mouse. Prostaglandin- and leukotriene-independent induction of infiltration by IL-1 and tumor necrosis factor. J. Immunol. 141, 1670–1677 3261759

[B89] Serezani C. H. Aronoff D. M. Jancar S. Mancuso P. Peters-Golden M. (2005). Leukotrienes enhance the bactericidal activity of alveolar macrophages against *Klebsiella pneumoniae* through the activation of NADPH oxidase. Blood 106, 1067–1075 10.1182/blood-2004-08-3323 15718414 PMC1895163

[B90] Soubani A. O. (1996). Pulmonary complications of bone marrow transplantation. Chest 109, 1066–1077 10.1378/chest.109.4.1066 8635332

[B91] Soubani A. O. Miller K. B. Hassoun P. M. (1996). Pulmonary complications of bone marrow transplantation. Chest 109, 1066–1077 10.1378/chest.109.4.1066 8635332

[B92] Soubani A. O. Uberti J. P. (2007). Bronchiolitis obliterans following haematopoietic stem cell transplantation. Eur. Respir. J. 29, 1007–1019 10.1183/09031936.00052806 17470622

[B93] Subramanian K. K. Jia Y. Zhu D. Simms B. T. Jo H. Hattori H. (2007). Tumor suppressor PTEN is a physiologic suppressor of chemoattractant-mediated neutrophil functions. Blood 109, 4028–4037 10.1182/blood-2006-10-055319 17202315 PMC1874585

[B94] Tarling J. D. Lin H. S. Hsu S. (1987). Self-renewal of pulmonary alveolar macrophages: evidence from radiation chimera studies. J. Leukoc. Biol. 42, 443–446 3316460

[B95] Therriault B. L. (2010). Characterization of bacterial infections in allogeneic hematopoietic stem cell transplant recipients who received prophylactic levofloxacin with either penicillin or doxycycline. Mayo Clin. Proc. 85, 711–718 10.4065/mcp.2010.0006 20675508 PMC2912731

[B96] Tomita Y. (1994). Myelosuppressive conditioning is required to achieve engraftment of pluripotent stem cells contained in moderate doses of syngeneic bone marrow. Blood 83, 939–948 7906567

[B97] Tse W. (2008). New insights into cord blood stem cell transplantation. Curr. Opin. Hematol. 15, 279–284 10.1097/MOH.0b013e328304ae2c 18536563

[B98] Versluys A. B. (2010). Strong association between respiratory viral infection early after hematopoietic stem cell transplantation and the development of life-threatening acute and chronic alloimmune lung syndromes. Biol. Blood Marrow Transplant. 16, 782–791 10.1016/j.bbmt.2009.12.534 20060053 PMC7110441

[B99] Wang Q. (2004). Neutrophils in innate immunity. Semin. Respir. Crit. Care Med. 25, 33–41 10.1055/s-2004-822303 16088447

[B100] Whittle A. T. Davis M. Shovlin C. L. Ganly P. S. Haslett C. Greening A. P. (2001). Alveolar macrophage activity and the pulmonary complications of haematopoietic stem cell transplantation. Thorax 56, 941–946 10.1136/thorax.56.12.941 11713357 PMC1745976

[B101] Wiltschke C. Krainer M. Nanut M. Wagner A. Linkesch W. Zielinski C. C. (1995). In vivo administration of granulocyte-macrophage colony-stimulating factor and granulocyte colony-stimulating factor increases neutrophil oxidative burst activity. J. Interferon Cytokine Res. 15, 249–253 10.1089/jir.1995.15.249 7584671

[B102] Wingard J. R. Hsu J. Hiemenz J. W. (2010). Hematopoietic stem cell transplantation: an overview of infection risks and epidemiology. Infect. Dis. Clin. North Am. 24, 257–272 10.1016/j.idc.2010.01.010 20466269

[B103] Winston D. J. Territo M. C. Ho W. G. Miller M. J. Gale R. P. Golde D. W. (1982). Alveolar macrophage dysfunction in human bone marrow transplant recipients. Am. J. Med. 73, 859–866 10.1016/0002-9343(82)90777-X 6756138

[B104] Wright J. R. (2005). Immunoregulatory functions of surfactant proteins. Nat. Rev. Immunol. 5, 58–68 10.1038/nri1528 15630429

[B105] Yang S. (2000). Cyclophosphamide prevents systemic keratinocyte growth factor-induced up-regulation of surfactant protein A after allogeneic transplant in mice. Am. J. Respir. Crit. Care Med. 162, 1884–1890 10.1164/ajrccm.162.5.2002053 11069830

[B106] Yu H. (2000). Innate lung defenses and compromised *Pseudomonas aeruginosa* clearance in the malnourished mouse model of respiratory infections in cystic fibrosis. Infect. Immun. 68, 2142–2147 10.1128/IAI.68.4.2142-2147.2000 10722612 PMC97396

[B107] Zimmerli W. Zarth A. Gratwohl A. Speck B. (1991). Neutrophil function and pyogenic infections in bone marrow transplant recipients. Blood 77, 393–399 1845932

